# Supplementary material for: IFI207, a young and fast‐evolving protein, controls retroviral replication via the STING pathway
Source: mBio. 2024 Jun 11;15(7):e01209-24. doi: 10.1128/mbio.01209-24 (PMC11253629; doi:10.1128/mbio.01209-24)
Supplement: Supplemental Figures — Fig. S1–S12. [file mbio.01209-24-s0001.pdf]

```
           100|                                                                                                               208|
IFI207_BL6    QEAGPATPTSTTSHMLTSERGETSATQEETSTAQGETSTAQAGTSTAQAGTSTAQAGTSTAQ-------TKRKQITKSEGEKKKKLTQEQAQLSEPLGTDRKKDEDCLQTPLMAPP
IFI207_BALB   QEAGPATPTSTTSHMLTSERGETSATQEETSTAQAGTSTAQARTSTAQAGTSTAQ--------------TKRKQITKSEGGKKKKLTQEQAQLSEPLGTDRKKDEDCLQTPLMPPP
IFI207_A      QEAGPATPTSTTSHMLASERGETSATQEETSTAQARTSTDQ----------------------------TKRKQITKSEGGKKKKLTQEQAQLSEPLGTDRKKDEDCLQTPLMPPP
IFI207_PWK    QEAGPATPTSTTSHMLTSERGETSATQEETSTAQAGTSTAQARTSTAQARTSTAQEGTSTAQAGTSTAQTKRKQITKSEGGKKKKLTQEQAQLSEPLGTDRKKDEDCLQTPLMPPP
IFI207_SIN    QEAGPATPTSTTSHMLTSERGETSATQEETSTAQAGTSTAQEGTSTAQ---------------------TKRKQITKSEGGKKKKLTQEQAQLSEPLGTDRKKDEDCLQTPLMPPP
IFI207_CzII   QEAGPATPTSTTSHMLTSERGETSATQEETSTAQAETSTAQVGTSTAQAGTSTAQAGTSTAQ-------TKRKQITKSEGGKKKKLTQEQAQLSEPLGTDRKKDEDCLQTPLMPPP
IFI207_CIM    QEAGPATPTSTTSHMLTSERGETSATQEETSTAQARTSTAQARTSTAQ---------------------TKRKQITKSEGGKKKKLTQEQAQLSEPLRTDRKKDEDCLQTPLMPPP
IFI207_SPRET  QEAGPATPTSTTSHMLASERGETSTTQEETSTAQAGTSTAQEGTSTAQ---------------------TKRKQITKSEGGKKKKLTQEQAQLSEPLGTDRKK-EDCLQTPLMPPP
IFI207_Caroli QEAGPATPTSTTNHMLASEG-------GETSTAQAQTSTAQ----------------------------TKRKQITRSEGGKKKKLSQEQAQLPEPLGTDIKKDEDCLKTPLMPPP
IFI207_Pahari QEAGPATPTSTTSHMSASEG-------GETSTAQVGTSTAQ----------------------------TKRKQITKSEGGKKKKLAQEQAQLPETSGTDIKKDEDCLQTPHKPPP
Mnda_BL6      QEAGPATPTSTTSHMLASERGETSATQEETSTAQAGTSTAQAGTSTAQAGTSTAQ---------------KRKSMREEETGVKKSKAAKEPDQPPCCEEP---TAMCQSPILHSSS
Mnda_Rat      REPGPSRPSSTASHMIVSEG-------WETSTAQAETSTAQGEPSTAQ----------------------KRKSMSEGKTEVKKTKASERPDQPPCPEEA---TAKCLSPIPQTSS
IFI205_BL6    QEASPATPTSTTSHMLASERGKTSTTQEETSTAQ------------------------------------KRKGMSEEKTDVKKIKASGKADQPPCCEGP---TATCQSPISQVSS
IFI205_Caroli QEAGPATPTS-TSHMLGSERGETSETQEETSTAQAGTSTAQ-----------------------------KRKGMSEEKTEVKKTKASGKADQPPCCEEF---IATCQSPISQVSS
Mnda_OncTor   TAASVKGKTPPTTSNTAASDG------GETSTAQVSLRNSSGLAVHRAVSSASS----------------KRKSINKEKTRVRKTKQSEGPDHPPCPEKA---TASCQSPELQTSS
Mnda_PerLeu   QEAGSN-TPTSTTSNTVASDG------GETSTAQ------------------------------------KRKS-NREKAGVKKTKQSEGPDHPLCPEEA---TARCQSPELQTSS
MNDA_Human    QEVGPA-TPTSISGSTVKTPEAQ-----------------------------------------------KRKTTSLGKTETKKNKISGIKRSKKSEEQILPSCPAEASMSTIMDH

ApoSly_207L1  QEAVPARATSTTKHMNKSLLQSER---GETSKTQAMTFTAQEESATAQ---------------------KKRKQITKTEGGKKNKL-EEQAQLPETSGTNTKRDEDCVQTPLKPPP
ApoSly_207L2  QEAGPATATSTTKHMNKCLSQFER---EETSTAQAMTSTAQEESATAQ---------------------KKRKQITKTEGGKKNKL-EEQAQLPETSGTNTKGDEDCVQTPLKPPP
ApoSly_207L3  QEAGPARATSTTRHMNTSLLQSER---GETSTAQAMTSTAQEESATAQ---------------------KKKKQITKTEGGKKKKLTEEQAQLPEPSGTNIKKEEDCLQTPLKPPP
ApoSly_207L4  QEAGPPTTTSTSSHMLASER-------GKTYTAPAETSTAQVESSTAQ---------------------KKRKQITKTEGGKKKKLTEEQAHLPETSRTNVKKDVDCVQTRPKSPP
ApoSly_207L5  QEASPPTTTSTTSHMLASER-------GKTYTAPAKTSTAQVKSSTAQ---------------------KKRKQITKTEGGKKKKLTEEQAHLPETSRTNVKNDVDCVRTPPKSPP
```

Fig. S1. Amino acid sequence around the PYD-proximal repeat region in human MNDA and rodent IFI207, IFI205 and MNDA. Color codes for the repeat unit: green-polarized (T,S,Q), yellow-non-polarized (A,V,P,M), cyan-positive-charged (R,K), red-acidic (E,D), blue-aromatic (W,F,Y) and purple (G). Numbers on top indicate residue location in IFI207_C57BL/6J.

```
             505|                                                                                                                   797|
IFI207_BL6    CTTQLTQGAASGTGKAFSLPEVKASMKVQAPQVSSPTASMSILNPNA++++++++++ 11 - 25 ++++++++++CPVTASRALSAIPVPSATVHSSPSWTPRRGTVPKEPSREEGHHQGPKQVMVL
207_Caroli    CTTQLTQGAASGTGKAFSLPEVKVSMKVQAPQVSSPTASISILNLNA+++++ units of repeats+++++++CPVTASRALSATPVPSSTVHSSPSWTPRRGTVPKEPSREEGHHQGPKQVMVL
207_Pahari    CTTQLTQGTASGTGKALPLPEVKVSMKVQAPQGSSPTASMSILNPNA++++++++++ in Mus +++++++++++SPVTASRALSATPVLSATVHSNPSRTPRRGTVPKEPSREEGHHQGPKQVMVL
IFI203_BL6    CTTPLTQGAESSTGEALPLPKVKACTIFQAPRVSSPTASSSIMKPHATSSKTSSSLLDQHATSSTASSSRLLALKLSPVTASRALSAIPGLSETIHRSPSSAPRRGTVPKEPSREEGHHQGPKQVMVL
203ps_BL6     CTTPLTQGAESSTGEFLPLPKVKACTIFQAPRVSSPTASSSIMKPHA--------------TSSTASSSSLLALKLSPVTASRALSAIPGLSETIHRSPSR-PRRGTVPKEPSREEGRHQGRKQVMVL
203_Caroli    CTTPLTQGAESSTGKALPLPKVKACTIFQAPRVSSPTASSSIMKSHATSSKTSSSLLDQHATSSTASSSSLLDLKLSPVTASRALSAIPGLSETIHSSPSR-PRKGTVPKEPSREEGHHQGPKQVMVL
203_Rat       CATQLIQEAASSTGKALPLPKVEASRKVQASPMSSATTPRSLLAPHA-----------------------------------------ASSTATRYSSPSRAPRRRAVPREPSREEGHHQGPKQVMVL
203_OncTor    CTTQVSQGVSYGTRQALPYPKVRASQRVHAPQLPSATALEGLLAPRG--SLPTASSSLLHPQVSPATASS-------------ALQAPLVTPAT--------PRCGNLPKEPSKVKGHHRVPKEVVVL
IFI16_Human   SEDTISK-----MNDFMRMQILKEGSHFPGPFMTS-IGPAESHPHTPQMPP------------STPSSSFLTTLKP---------------------------RLKTEPEECSIEDSAQSDLKEVMVL

ApoSly_207L1  CSTQLTQGAASCTRKDLSLPKVTSSRNVQASQLSSATASSSILVPYAISSTASSSLLAPHA--TPSTATINLALQLSPATASRALHAASGLSATVYSSPTRTPRKEIVPTEPTKEEGHQKGPKQVMVL
ApoSly_207L2  CTTRLTQGATSCTGKELSLPKVTSFRNVQASQVCSATASSSTLVPYAISSTASSSLLAPHA--TPSTATINLALQLSPATASRALHAAPWLSATVHSSPTRTPRRGIVPTEPTKEEGHQKGPKQVMVL
ApoSly_207L3  CSTQLTQGEACVCGKDLSLPKVTSSRNVQAPHMSSATQSSSLLVPDTTSSTASSSLLASHA-ALPTTCSRFSPPHLSQATASITHSAIPGPSATVHSSKSKTPKRGIVPKEPAKEEGHQKGPKQVMVL
ApoSly_207L4  (exon destructed by a long insertion)
ApoSly_207L5  (exon destructed by a long insertion)
```

Fig. S2. Amino acid sequence around the HIN-proximal repeat region in human IFI16 and rodent IFI203 and IFI207. Rodent IFI203 has 1-2 copies of the HIN-proximal repeat sequence, and human IFI16 also has one copy. Color codes for the repeat unit: green-polarized (T,S,Q), yellow-non-polarized (F,L,A,P,V), cyan-basic (H,K), red-acidic (E,D), and blue-aromatic (F,Y). Numbers on top indicates residue location in IFI207_C57BL/6J.

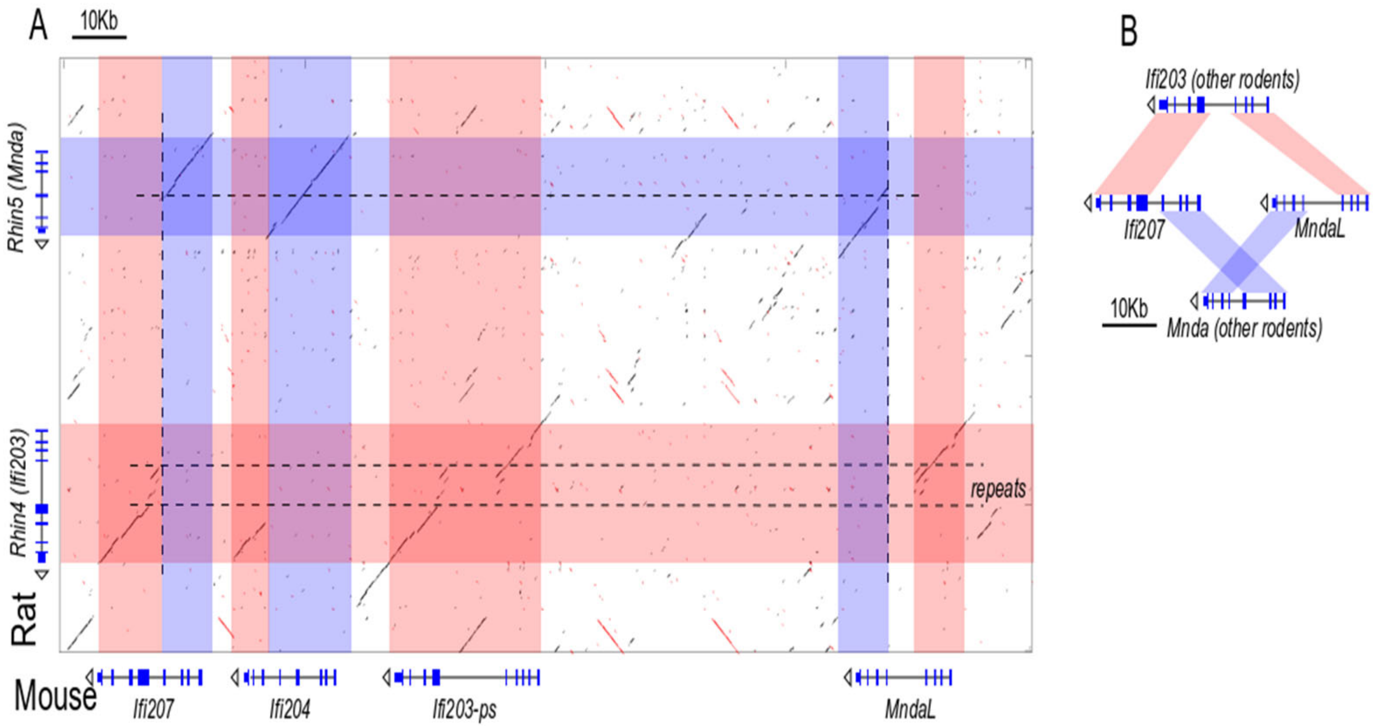

Fig. S3. *Ifi207* and *MndaL* in the *Mus* Genus are possibly created by recombination between ancestral *Ifi203* and *Mnda* genes. A) Using the rat genome, which is similar to most other rodent species, as an example in the dot plot, the structure of mouse *Ifi207*, *Ifi204* and *MndaL* appear to be the result of recombinations between rat *Mnda* (*Rhin5*, indicated with blue shades) and *Ifi203 homologue* (*Rhin4*, red shades). B) Broken at a repeat rich region, the ancestral *Ifi203* and *Mnda* fused into mice *Ifi207* and *MndaL*.

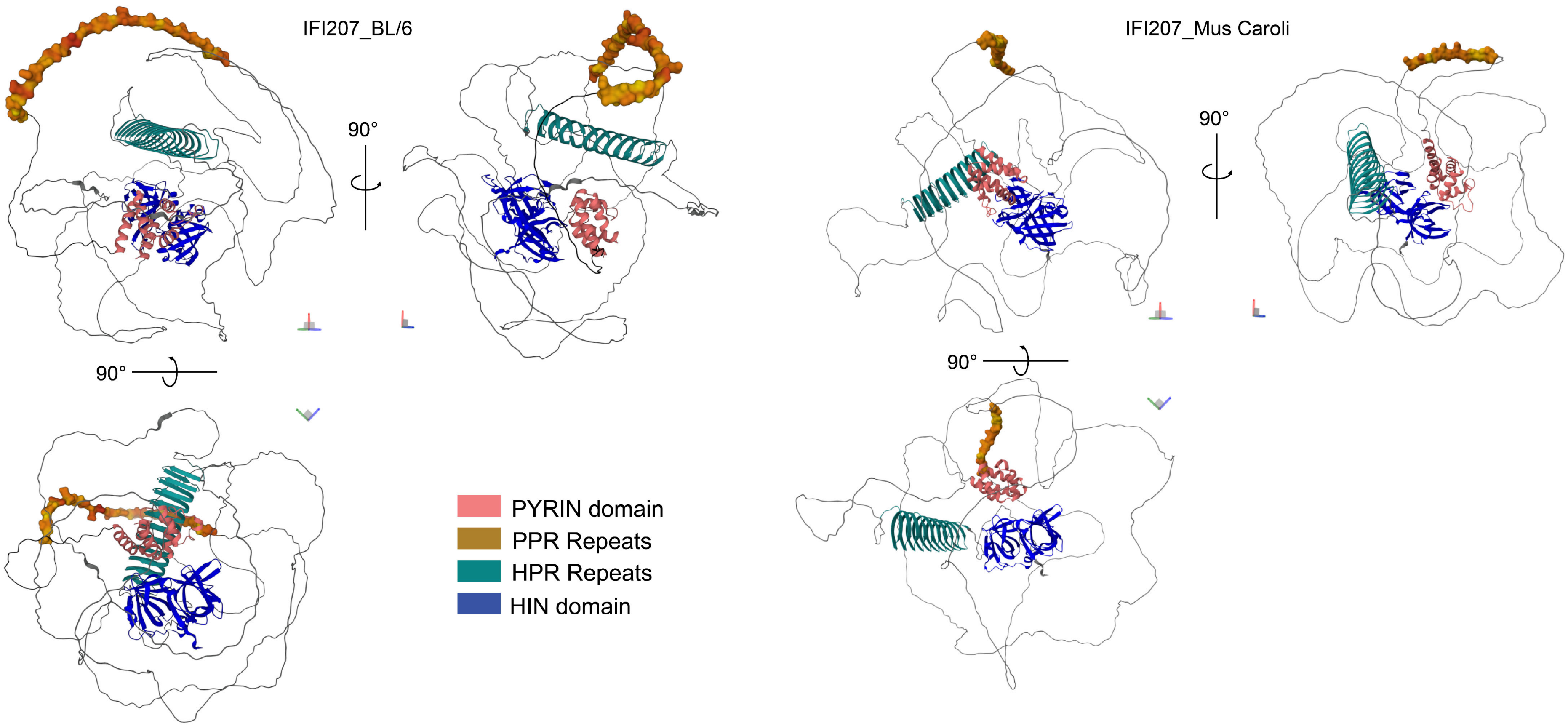

Fig. S4. Predicted structure of *M. caroli* IFI207. Comparison of C57BL/6 (E9Q3L4) and *M. caroli* (A0A6P7R030) IFI207 predicted protein structures. Domains are colored as follows: PYD domain (pink), PYD-proximal repeat (PPR) (brown), HIN-proximal repeat (HPR) (teal), HINB (blue). Structures were downloaded from AlphaFold (https://alphafold.ebi.ac.uk/).

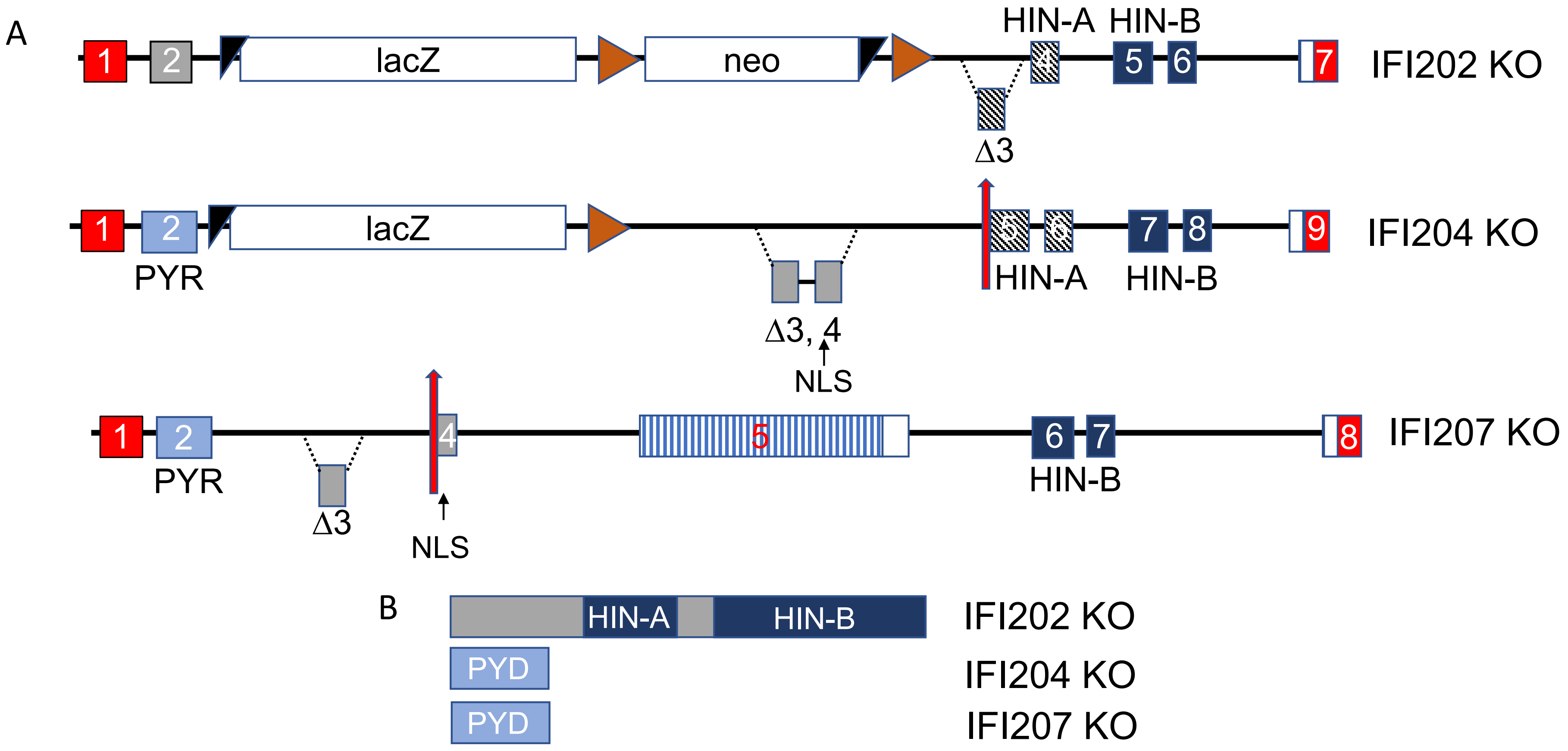

Fig. S5. A) Diagram of the mouse knockout alleles. Not drawn to scale. Exon 3 in the *Ifi202* gene was replaced with a lacZ/neo cassette. This results in a protein lacking exon 3. A similar lacZ/neo cassette with flox sites upstream and downstream of exons 3 and 4 was engineered into the *Ifi204* locus; crossing the parental mice with CMVcre mice resulted in the deletion of these exons and the introduction of a stop codon at the beginning of exon 5 (red arrow). Details of the construction of the parental *Ifi202* and *Ifi204* mouse knockouts can be found on the KOMP Repository website. CRISPR/Cas9 was used to delete exon 3 of the *Ifi207* gene, as described in Methods. This causes a stop codon at the beginning of exon 4. Red boxes depict non-coding exons. Striped sequences in exon 5 of *Ifi207* represent the repeat region. NLS, nuclear localization signal. B) Predicted proteins from the KO alleles.

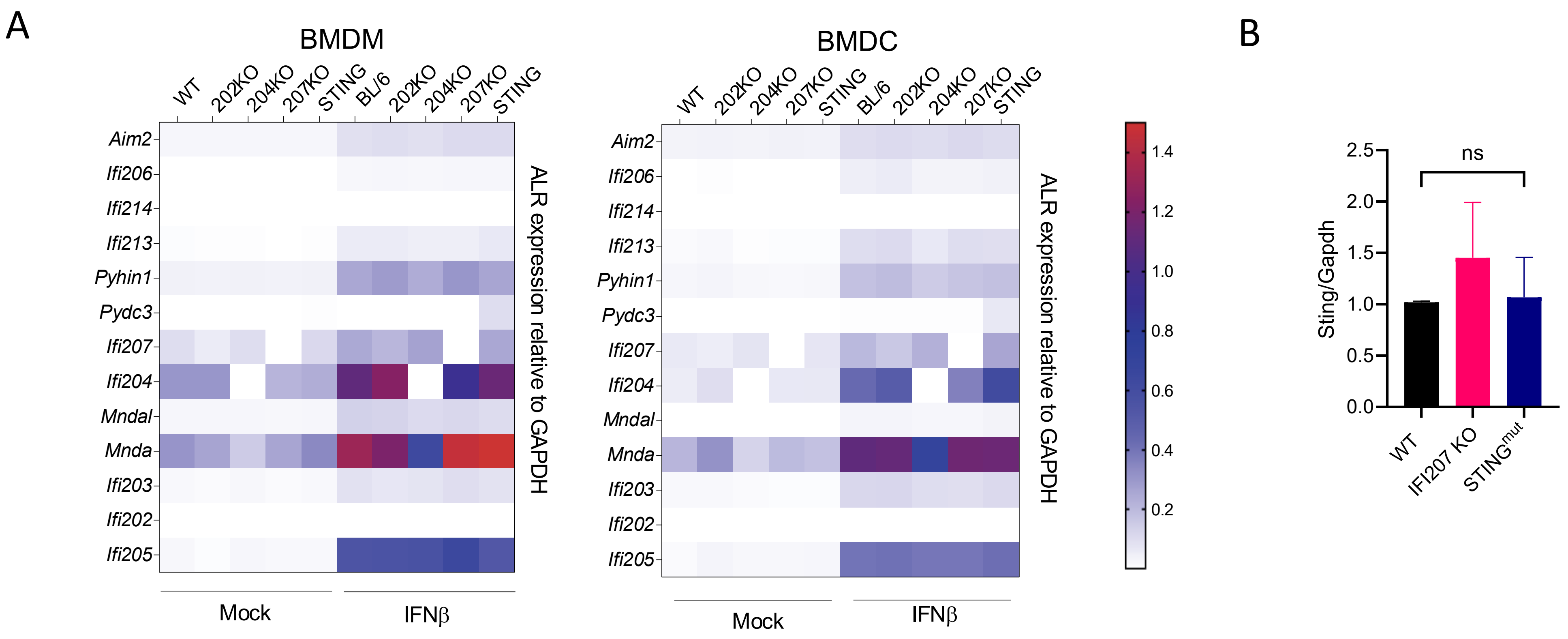

Fig. S6. *Ifi207* expression in BMDMs and BMDCs. BMDMs and BMDCs from the indicated mice were treated with or without mouse IFNβ for 4h. mRNA expression was quantified by RT-qPCR. Means from 2 independent experiments performed with duplicate or triplicate samples per treatment were plotted. B) *Sting* RNA was isolated from BMDMs from the indicated mice and RT-qPCR was used to analyze expression levels. Shown is the average of 3 independent experiments ± SD. One-way ANOVA was used to determine significance. ns, not significant.

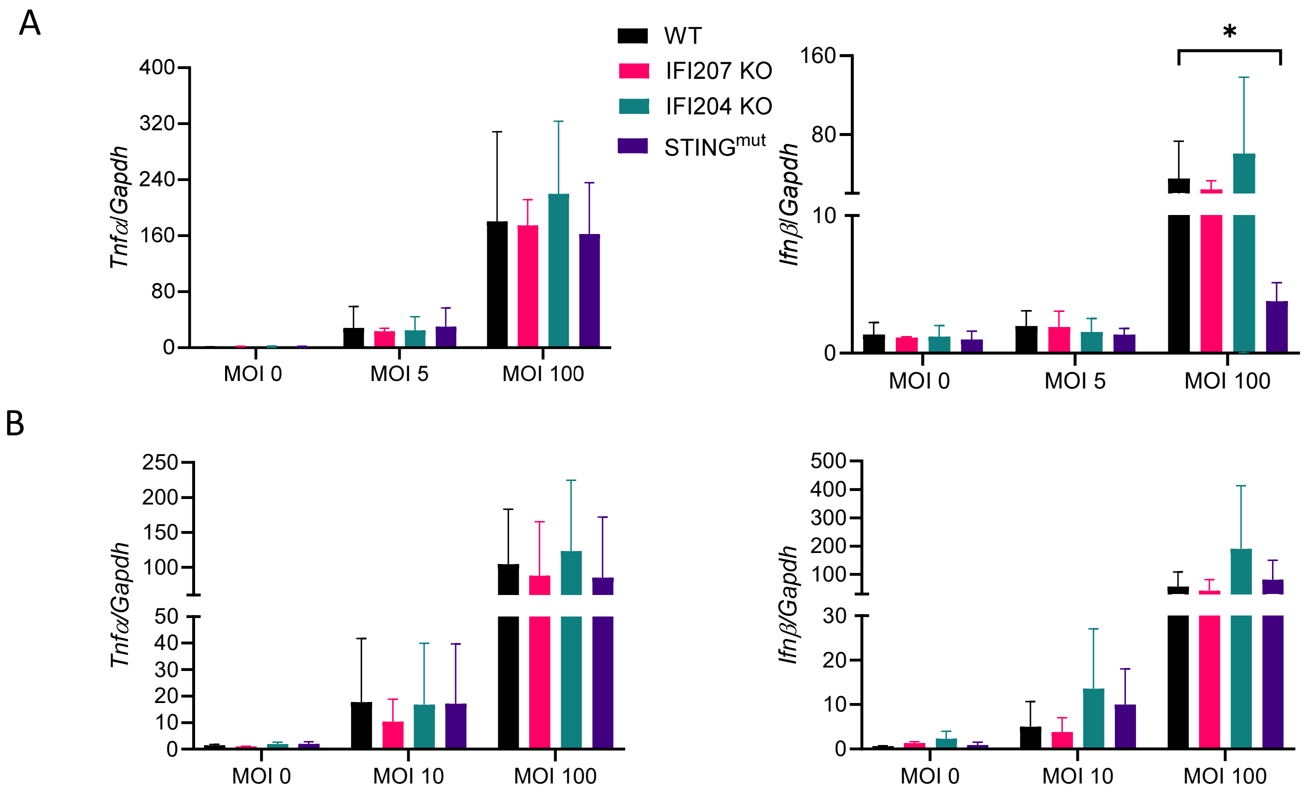

Fig. S7. IFI207 does not contribute to the response to bacteria. A) Primary BMDMs from mice of the indicated genotypes were infected with *S. aureus* at the indicated MOI. RNA was analyzed by RT-qPCR for *Tnf*α and *Ifn*β at 4 hr post-infection. B) BMDMs were infected with *K. pneumoniae* at the indicated MOI. RNA was analyzed by RT-qPCR for *Tnf*α and *Ifn*β at 6 hr post-infection. Shown are the averages ± the SD of 3 independent experiments. Statistical significance was determined by unpaired T test. * $P \leq 0.01$.

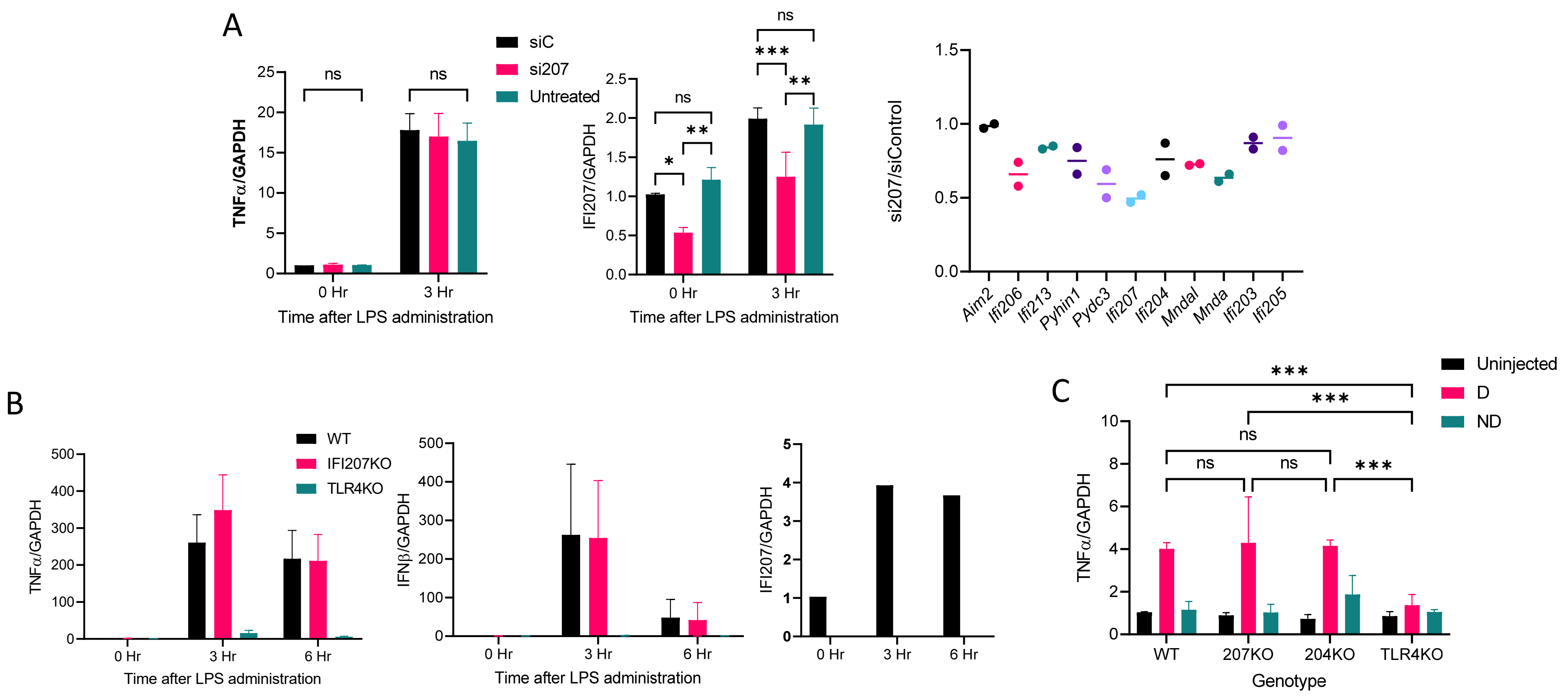

Fig. S8. IFI207 does not contribute to the LPS response. A) NR9456 cells were transfected with the indicated siRNAs and treated with 10ng/ml LPS for 3 hr. RNA was analyzed by RT-qPCR for *Tnfα* and *Ifi207*. Knockdown of IFI207 is shown in the right panel. Right panel: IFI207 siRNAs largely target *Ifi207*. Knockdown efficiency was determined by RT-qPCR with primers specific for the indicated genes. Duplicate experiments were performed; both values are shown.B) Primary BMDMs from mice of the indicated genotypes were treated with 10ng/ml LPS for the indicated times. RNA was analyzed by RT-qPCR for*Tnfα*, *Ifnβ* and *Ifi207*. No IFI207 RNA was detected in KO fibroblasts (panel B). C) WT, IFI207, IFI204 and TLR4 KO mice were injected into the right hind footpad of with 1μg of LPS. At 2 hr after LPS injection, lymphocytes were isolated from the popliteal draining (D) and non-draining (ND) lymph nodes. RNA was isolated, and *Tnfα* RNA expression was analyzed by RT-qPCR. Uninjected lymph nodes were used as controls.

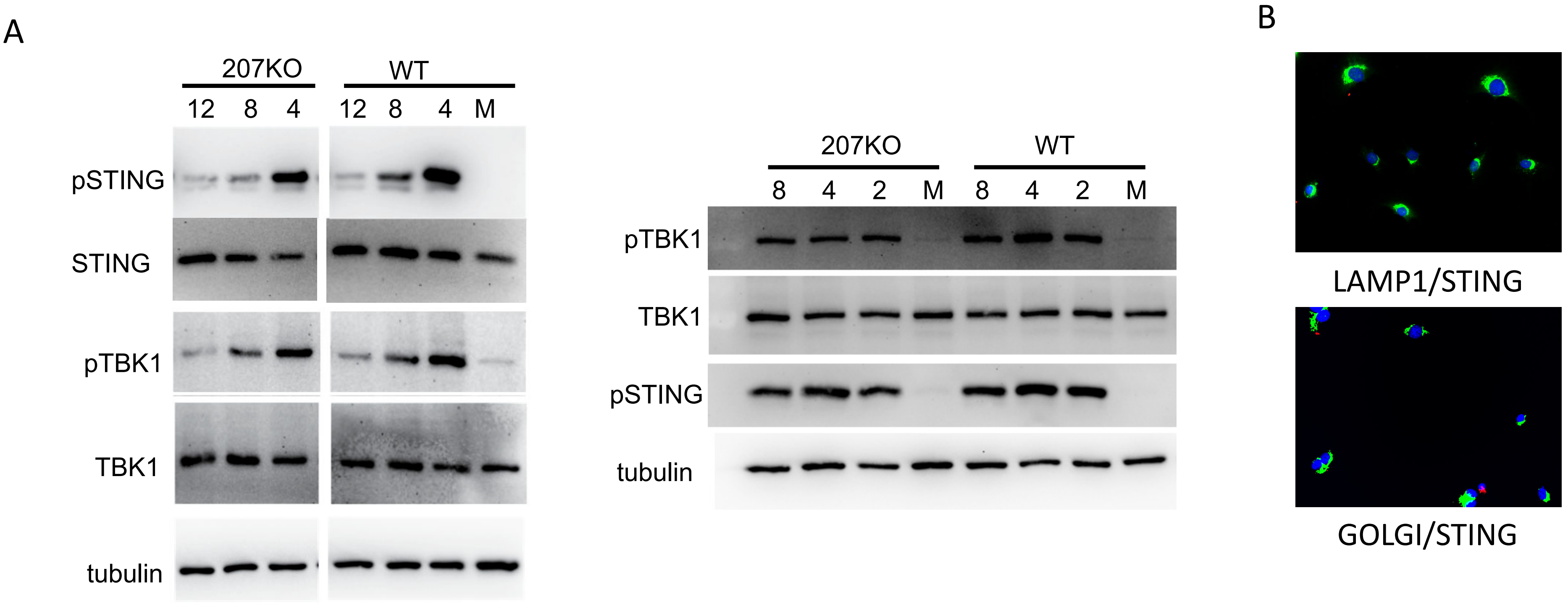

Fig. S9. IFI207 stabilizes STING. A) BMDCs were transfected with ISD and at 4, 8 and 12 or 2, 4 and 8 hr post-transfection, protein expression was analyzed by western blot with the indicated antibodies. Blots were probed with anti-tubulin to control for loading. Two independent experiments are presented. Abbreviations: M, mock-transfected. B) No STING was detected in fibroblasts from STING^{mut} mice. See legend to Figure 2 for details.

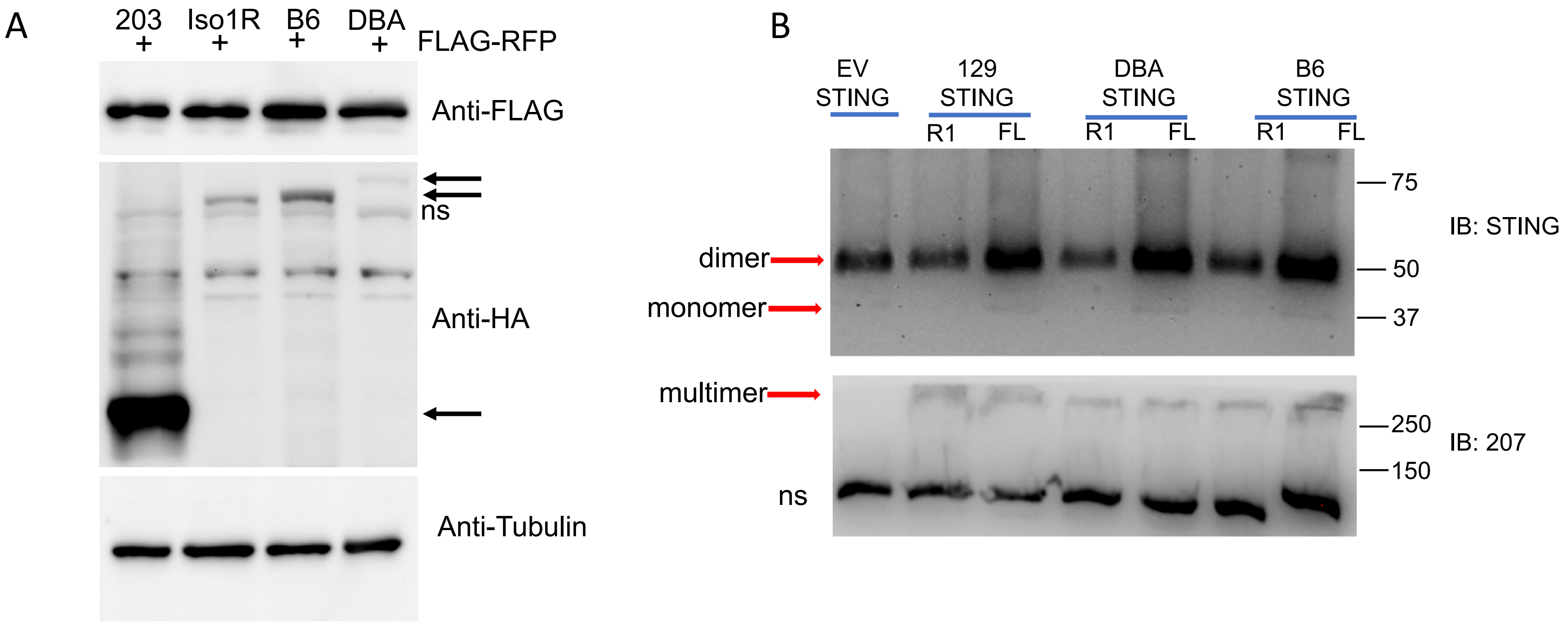

Fig. S10. IFI207 stabilizes STING. A) IFI207 does not stabilize FLAG-RFP. 293T cells were co-transfected with an RFP-FLAG and IFI203Iso2, IFI203 Iso1R, and the BL/6 and DBA IFI207 HA-tagged expression plasmids. Western blots were performed with the indicated antibodies. ns, non-specific. B) Full-length IFI207 increases STING dimers. 293T cells were co-transfected with HA-tagged IFI207 and FLAG-tagged STING expression plasmids. Lysates were run on native gels and protein expression was examined by western blot with the anti-HA or -FLAG antibodies. FL, Full length IFI207; R1, Repeat mutant IFI207; EV, empty vector; ns, non-specific.

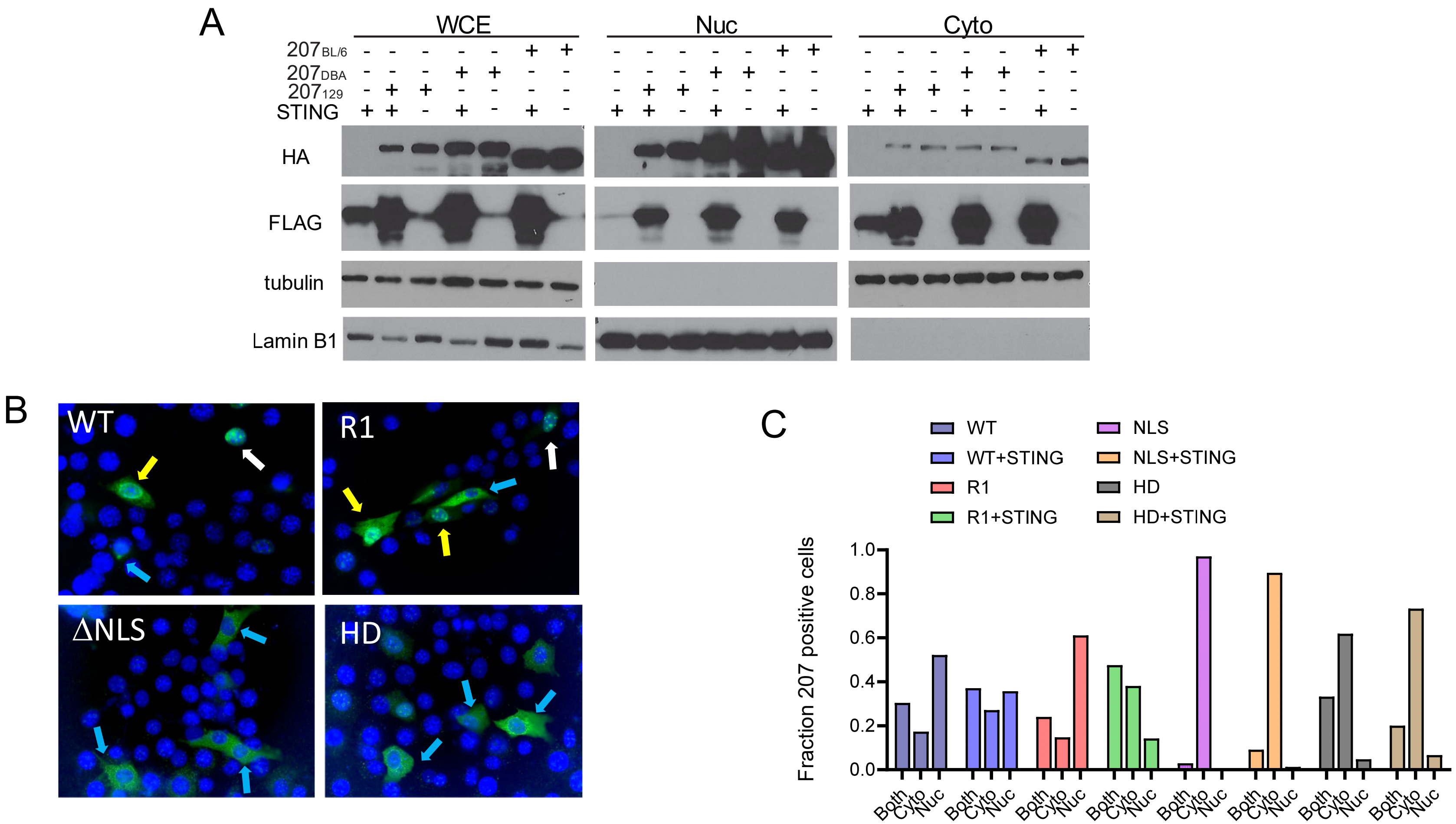

Fig. S11. Subcellular localization of STING and IFI207. (A) HEK293T cells were transfected with full length HA-tagged IFI207 expression constructs with or without FLAG-tegged STING expression constructs. Cell lysates were fractionated and proteins were analyzed by western blotting with the indicated antibodies. WCE, whole cell extract; Cyto, cytoplasm; Nuc, nucleus. B) Localization of IFI207 in the absence of STING. NIH3T3 cells were transfected with the indicated HA-tagged IFI207 expression vectors. White arrow: nuclear, blue arrow: cytoplasm, yellow arrow, both. C) Localization of IFI207 in the presence and absence of STING co-transfection. See Fig. 4I for details. Five to 30 fields of each transfection were scored.

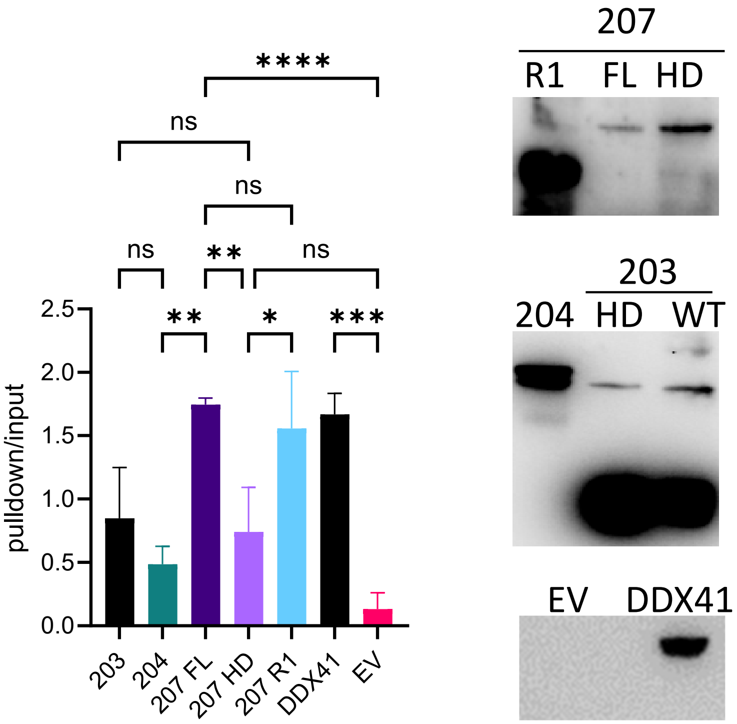

Fig. S12. IFI207 RI mutant binds reverse transcripts. A) DNA pulldown assays with extracts from cells transfected with the indicated constructs. Anti-HA was used for the IFI constructs, and anti-myc was used for DDX41. Strong stop early reverse transcript primers were used to carry out RT-qPCR. Average of 3 independent experiments with S.D. *, $p \leq 0.02$; **, $p \leq 0.008$; ***, $p \leq 0.0002$; ****, $p \leq 0.0001$ ‡, $p \leq 0.07$ (two-tailed T test). Right panel: Extracts from 293T cells transiently transfected with the indicated expression vectors were immunoprecipitated with anti-HA antibodies and analyzed on western blots. Shown are the results of a single experiment (representative of 3 independent experiments)
