# Supplementary material for: IFI207, a young and fast‐evolving protein, controls retroviral replication via the STING pathway
Source: mBio. 2024 Jun 11;15(7):e01209-24. doi: 10.1128/mbio.01209-24 (PMC11253629; doi:10.1128/mbio.01209-24)
Supplement: Table S1 — Assemblies. [file mbio.01209-24-s0002.docx]

| Table S1. Species analyzed for the presence of *Ifi207* in Glires clade. | | | |  |  |  |  |  |  |  |  |
| --- | --- | --- | --- | --- | --- | --- | --- | --- | --- | --- | --- |
| Species | Tribe | Sub family | Family | Super family | Suborder | Order | Assembly | Release time | Level | Quality | *Ifi207* |
| Mus musculus | Murini | Murinae | Muridae | Muroidea | Myomorpha | Rodentia | GRCm39 | 2021 | Chr | Refence Genome | Yes |
| Mus Spretus | Murini | Murinae | Muridae | Muroidea | Myomorpha | Rodentia | MGP | 2022 | Chr | VGP * | Yes |
| Mus Spicilegus | Murini | Murinae | Muridae | Muroidea | Myomorpha | Rodentia | UCSF_Mcou_1 | 2019 | Chr | Illumina 73x | Yes |
| Mus Caroli | Murini | Murinae | Muridae | Muroidea | Myomorpha | Rodentia | MGP | 2022 | Chr | VGP * | Yes |
| Mus Pahari | Murini | Murinae | Muridae | Muroidea | Myomorpha | Rodentia | PAHARI_EIJ_v1.1 | 2017 | Chr | WGS 74x | Yes |
| Mastomys coucha | Praomyini | Murinae | Muridae | Muroidea | Myomorpha | Rodentia | UCSF_Mcou_1 | 2019 | Chr | Illumina 73x | No |
| Rattus norvegicus | Rattini | Murinae | Muridae | Muroidea | Myomorpha | Rodentia | RN6 | 2014 | Chr | Refence Genome | No |
| Rattus rattus | Rattini | Murinae | Muridae | Muroidea | Myomorpha | Rodentia | Rrattus_CSIRO_v1 | 2020 | Chr | Illumina + ONT 130x | No |
| Grammomys surdaster | Arvicanthini | Murinae | Muridae | Muroidea | Myomorpha | Rodentia | NIH_TR_1.0 | 2019 | Scf | Illumina 50x | No |
| Arvicanthis niloticus | Arvicanthini | Murinae | Muridae | Muroidea | Myomorpha | Rodentia | mArvNil1.pat.X | 2020 | Chr | VGP * | No |
| Apodemus speciosus | Apodemini | Murinae | Muridae | Muroidea | Myomorpha | Rodentia | Aspe_assembly01 | 2017 | Scf | Illumina 65x | No |
| Apodemus sylvaticus | Apodemini | Murinae | Muridae | Muroidea | Myomorpha | Rodentia | MApoSyl1.1 | 2020 | Chr | VGP * | Yes ** |
| Tokudaia osimensis | Apodemini | Murinae | Muridae | Muroidea | Myomorpha | Rodentia | T_osimensis_1.0 | 2022 | Scf | Illumina 170x | No |
| Pachyuromys duprasi | Desmodilliscini | Gerbillinae | Muridae | Muroidea | Myomorpha | Rodentia | Pachyuromys_duprasi_HiC | 2023 | Chr | Dnazoo | No |
| Psammomys obesus | Gerbillini | Gerbillinae | Muridae | Muroidea | Myomorpha | Rodentia | mPsaObe1.curated_primary_1811 | 2021 | Scf | Illumina +10x | No |
| Lophiomys imhausi |  | Lophiomyinae | Muridae | Muroidea | Myomorpha | Rodentia | mLopImh1.curated_primary_1811 | 2021 | Scf | Illumina +10x | No |
| Acomys russatus | - | Deomyinae | Muridae | Muroidea | Myomorpha | Rodentia | mAcoRus1.1 | 2020 | Chr | VGP * | No |
| Acomys kempi | - | Deomyinae | Muridae | Muroidea | Myomorpha | Rodentia | mAcoKem2_REL_1905 | 2021 | Chr | PacBio + Illumina | No |
| Peromyscus Leucopus | Reithrodontomyini | Neotominae | Cricetidae | Muroidea | Myomorpha | Rodentia | UCI_PerLeu_2.1 | 2020 | Chr | PacBio + Illumina + HiC | No |
| Peromyscus maniculatus | Reithrodontomyini | Neotominae | Cricetidae | Muroidea | Myomorpha | Rodentia | HU_Pman_2.1.3 | 2020 | Chr | Illumina 115x | No |
| Onychomys torridus | Reithrodontomyini | Neotominae | Cricetidae | Muroidea | Myomorpha | Rodentia | mOncTor1.1 | 2020 | Chr | VGP * | No |
| Cricetulus griseus | - | Cricetinae | Cricetidae | Muroidea | Myomorpha | Rodentia | CHOK1S_HZDv1 | 2017 | Scf | WGS 74x | No |
| Mesocricetus auratus | - | Cricetinae | Cricetidae | Muroidea | Myomorpha | Rodentia | BCM_Maur_2.0 | 2021 | Scf | ONT 88x | No |
| Jaculus jaculus |  | Dipodinae | Dipodidae | Dipodoidea | Myomorpha | Rodentia | mJacJac1.mat.Y.cur | 2021 | Chr | VGP * | No |
| Sciurus vulgaris | Sciurini | Sciurinae | Sciuridae | - | Sciuromorpha | Rodentia | mSciVul1.2 | 2020 | Chr | VGP * | No |
| Sciurus carolinensis | Sciurini | Sciurinae | Sciuridae | - | Sciuromorpha | Rodentia | mSciCar1.2 | 2020 | Chr | VGP * | No |
| Perognathus longimembris | Perognathus | Perognathinae | Heteromyidae | Geomyoidea | Castorimorpha | Rodentia | ASM2315922v1 | 2022 | Chr | PacBio 23x | No |
| Oryctolagus cuniculus | - | - | Leporidae | - | - | Lagomorpha | UM_NZW_1.0 | 2021 | Chr | BacBio 40x | No |
| Ochotona Princeps | - | - | Ochotonidae | - | - | Lagomorpha | OchPri4.0 | 2020 | Chr | Illumina + Dovetail | No |
