# Supplementary material for: IFI207, a young and fast‐evolving protein, controls retroviral replication via the STING pathway
Source: mBio. 2024 Jun 11;15(7):e01209-24. doi: 10.1128/mbio.01209-24 (PMC11253629; doi:10.1128/mbio.01209-24)
Supplement: Supplemental Tables — Tables S4–S8. [file mbio.01209-24-s0005.docx]

**Table S4.** Guide RNAs used to generate IFI207 knockouts (A) and (B) PCR primers used to genotype IFI207, IFI204 and IFI202 knockout mice. IFI202 knockout mice were genotyped by the presence of LacZ sequences and the absence of exon 3.

A)

| Sequence | Chr | Chr Start | Chr End |
| --- | --- | --- | --- |
| TAAGTGGTTTCGTCAAATAAAGG | **1** | 173734772 | 173734794 |
| GTGTAAATATGCTACAACCATGG | **1** | 173734874 | 173734896 |
| TAACTTTCTAAAGTCAATTGTGG | **1** | 173735725 | 173735747 |
| CCTTCTTAAGACTCTATTTAATC | **1** | 173735813 | 173735835 |

B)

| Primer Name | Primer Sequence (5’>3’) |
| --- | --- |
| Ifi207_DF1 | TCAGTTGAGCAAAGTGCGTC |
| Ifi207_ER1 | GCAGACAGGCAGAGGTGATT |
| Ifi207_EF1 | TGATTTGGTCATGTTTTGTGCAT |
| Ifi207_DR1 | TCCAGATGTAACATAGAGGTTCG |
| Ifi204F | CCAGCTGATTCTGGATTGGGCAAACTG |
| Ifi204R | CCTCTGGGAATGTTCTGATTCTGGGG |
| Ifi202F ex3 | CAGTGGCTACAGAGAGCCAA |
| Ifi202R ex3 | TAGGAGCAGCCTCTGACACA |
| LacZ forward | GGCCGTCGTTTTACAACGTCGTCGTG |
| LacZ reverse | GCGAGTAACAACCCGTCGGATTC |

**Table S5.** Genbank accession numbers for sequences.

| Mouse Strain | Source | Description | GenBank accession # |
| --- | --- | --- | --- |
| DBA/2J | cDNA | Full length cDNA | ON550581 |
| 129P2/OlaHsd | cDNA | Full length cDNA | ON550582 |
| C57BL/6N | cDNA | Repeat region | ON550583 |
| DBA2J | cDNA | Repeat region | ON550584 |
| Aim2-/- (B6.129P2-Aim2Gt(CSG445)Byg/J) | cDNA | Repeat region | ON550585 |
| 129P2/OlaHsd | cDNA | Repeat region | ON550586 |
| BALB/cJ | cDNA | Repeat region | ON550587 |
| M. M. castaneus | cDNA | Repeat region | ON550588 |

**Table S6**. Primers used for expression analysis (Fig. 2) and MLV reverse transcript pulldowns (Fig. S8). Primers used for *Alr* expression analysis were described in Brunette et al. (*1*).

| **Target** | **Forward (5’-3’)** | **Reverse (5’-3’)** |
| --- | --- | --- |
| Aim2 | GTTGAATCTAACCACGAAGTCC | CTACAAGGTCCAGATTTCAACTG |
| Ifi206 (PyblhinC) | CGACAGCAATGTGATGAATATCC | TTCCTAGCCTGGATGACCTGC |
| Ifi214 (PyhinB) | CCAACAAGATCATCCAGCAGG | TTCTCTCCTTTCATGTCATATGC |
| Ifi213 (Pydc4) | CCTCTGAACCCTGTCTGCCA | CCAGCATCCTCTGGGAACTTT |
| Pyhin1 | TCTGGACCCTCCAGTGTCTT | ACCTTGCTGGTGACCATTTT |
| Pydc3 | GCCTGATGGAAGCTTGGGAA | CTGGGGAGTCAGTGGTTCAC |
| Ifi207 | CTTCTACAGCCCAGACAAAGAG | TCGGTTCCTAAAGGTTCTGAAAG |
| Ifi204 | TGTGTGGAGAACACAGTTTCATC | GAGATAACCATTGTTGGATTAACG |
| Mndal | ACCCAGCAGTTCCTTAAACAAG | TTCAATCATTTGGTCAGGATCAG |
| Mnda | TCTGAAAGAGGCGAGACTTCTG | TCTGGGAATGTTCTGGTTCTGG |
| Ifi203 | ATGTCAGGTGTGAACCAGGC | TACAGACCTCAGGAGGCACA |
| Ifi202 | AGCTGACACACTCTGCCTTG | TGATTGAGTTCAAGCCGGGA |
| Ifi205 | AGCATCTGAAAGAGGCAAGAC | GAGGCTGATCTGCTTTCCCA |
| MLV SSS | CCTCCGATTGACTGAGTCGCCCC | ATGAAAGACCCCCGCTGACGG |
| IFNb | AAGAGTTACACTGCCTTTGCCACT | CACTGTCTGCTGGTGGAGTTCATC |
| actin | TGGAATCCTGTGGCATCCATGAAAC | TAAAACGCAGCTCAGTAACAGTCCG |

**Table S7.** Sequencing, cloning and mutagenesis primers.

| **Target** | **Forward (5’-3’)** | **Reverse (5’-3’)** |
| --- | --- | --- |
| Ifi207 repeat region | GTAAAAGCATCCATGAAAGTCCAAGC | GGATGGACTGCTGTGTACTGTTGC |
| Full-length IFI207_DBA_ and IFI207_129_ | TGGTGGGAATTCAAGATGGTGAAAGAATATAAAAGAATTGTTCTG | TGGTGGCTCGAGTCAAGCGTAATCTGGAACATCGTATGGGTACCTTCTAGCATTGATGACCTG |
| Ifi207 cDNA | TGGTGGGAATTCAAGATGGTGAAAGAATATAAAAGAATTGTTCTG | TGGTGGCTCGAGTCAAGCGTAATCTGGAACATCGTATGGGTACCTTCTAGCATTGATGACCTG |
| ΔPYD | GTAACAGGAGAAACATCAC | CATCTTGAATTCCACCAC |
| R1 | GTATCCTGAATCCACAGTTGTGTCCAGTAGCAGC | TCATGGATGCTGTTGGGGAAGACACCTGAGGTGCTTGG |
| ΔNLS | CAAATCACAAAAAGTGAAGGTGG | TGTCTGGGCTGTGGAAGT |
| HD | AACAGAACCAGCTACAGCCGACCTGGAAG | ACTTTCAGCACCATCACTTG |
| HIN | TGGTGGGAATTCAAGATGACTGTACCAAAGGAGCCTTC | TGGTGGCTCGAGTCACGTAGAATCGAGACCGAGGAGAGGGTTAGGGATAGGCTTACCCCTTCTAGCATTGATGACCTG |

**Table S8.** Cloning and Sequencing primers for IFI203-Iso1 and Iso1-207(repeat) constructs.

| 203F | ATGATGGCTGAATACAAGAATATTG |
| --- | --- |
| 203R | GAAGTATGGTTCCAGAGAGTTTC |
| B6 203 1F | GCACCAAGGAGAGGAACTGTACC |
| B6 203 2R | CTTGTTTGAGGAACTGCTGGATGG |
| B6 203 3F | AGTTCCTCAAACAAGAAGAATCCTGTTGTGGCGTCC |
| B6 203 4R | TCCTCTCCTTGGTGCGCTGGATGGACTTCTGTGTATTG |
| 207 21bpF | CTGGATCAACATGCAAGTATCCTGAATCCAAATGCAACCC |
| b6 21bpR | TGCTGTTGATGAAGTCCTAGATGCTGTTACTGGACAC |
| 203 VF | ACTTCATCAACAGCATCCAGCAGC |
| 203 VB | TGCATGTTGATCCAGGAGACTGCTG |
| 203F1751 | CATCCAGCAGTATCATGAAGC |

1. R. L. Brunette *et al.*, Extensive evolutionary and functional diversity among mammalian AIM2-like receptors. *J. Exp. Med.* **209**, 1969-1983 (2012).
